# Detection of maternal and fetal stress from the electrocardiogram with self-supervised representation learning

**DOI:** 10.1038/s41598-021-03376-8

**Published:** 2021-12-17

**Authors:** Pritam Sarkar, Silvia Lobmaier, Bibiana Fabre, Diego González, Alexander Mueller, Martin G. Frasch, Marta C. Antonelli, Ali Etemad

**Affiliations:** 1 Department of ECE and Ingenuity Labs Research Institute, Queen’s University, Kingston, ON Canada; 2 Department of Obstetrics and Gynecology, Technical University of Munich, Munich, Germany; 3 Department of Obstetrics and Gynaecology, University of Washington, Seattle, WA USA; 4 Center on Human Development and Disability, University of Washington, Seattle, WA USA; 5 Facultad de Farmacia y Bioquímica, Universidad de Buenos Aires, Buenos Aires, Argentina; 6 Department of Cardiology, Technical University of Munich, Munich, Germany; 7 IBCN, Facultad de Medicina, Universidad de Buenos Aires, Buenos Aires, Argentina

**Keywords:** Development of the nervous system, Biomarkers, Paediatric research, Translational research, Biomedical engineering, Scientific data

## Abstract

In the pregnant mother and her fetus, chronic prenatal stress results in entrainment of the fetal heartbeat by the maternal heartbeat, quantified by the fetal stress index (FSI). Deep learning (DL) is capable of pattern detection in complex medical data with high accuracy in noisy real-life environments, but little is known about DL’s utility in non-invasive biometric monitoring during pregnancy. A recently established self-supervised learning (SSL) approach to DL provides emotional recognition from electrocardiogram (ECG). We hypothesized that SSL will identify chronically stressed mother-fetus dyads from the raw maternal abdominal electrocardiograms (aECG), containing fetal and maternal ECG. Chronically stressed mothers and controls matched at enrolment at 32 weeks of gestation were studied. We validated the chronic stress exposure by psychological inventory, maternal hair cortisol and FSI. We tested two variants of SSL architecture, one trained on the generic ECG features for emotional recognition obtained from public datasets and another transfer-learned on a subset of our data. Our DL models accurately detect the chronic stress exposure group (AUROC = 0.982 ± 0.002), the individual psychological stress score (R2 = 0.943 ± 0.009) and FSI at 34 weeks of gestation (R2 = 0.946 ± 0.013), as well as the maternal hair cortisol at birth reflecting chronic stress exposure (0.931 ± 0.006). The best performance was achieved with the DL model trained on the public dataset and using maternal ECG alone. The present DL approach provides a novel source of physiological insights into complex multi-modal relationships between different regulatory systems exposed to chronic stress. The final DL model can be deployed in low-cost regular ECG biosensors as a simple, ubiquitous early stress detection and monitoring tool during pregnancy. This discovery should enable early behavioral interventions.

## Introduction

Maternal chronic stress during pregnancy programs the fetal brain for altered developmental trajectories. We showed that in stressed mother-fetus dyads, this results in measurable synchronization of the fetal heartbeat by the maternal heartbeat, quantified by the fetal stress index (FSI)^1^. Can this biophysical phenomenon be scaled to an easily deployable biomarker of chronic stress in pregnant mothers to help guide early interventions which can reverse altered fetal developmental trajectories?

Deep learning (DL)-based approaches^2^ to pattern detection in complex physiological data have shown high accuracy in noisy real-life environments^3,4^. Nonetheless, little is known about their utility in the setting of non-invasive biometrics obtained during human pregnancy.

**Figure 1:**
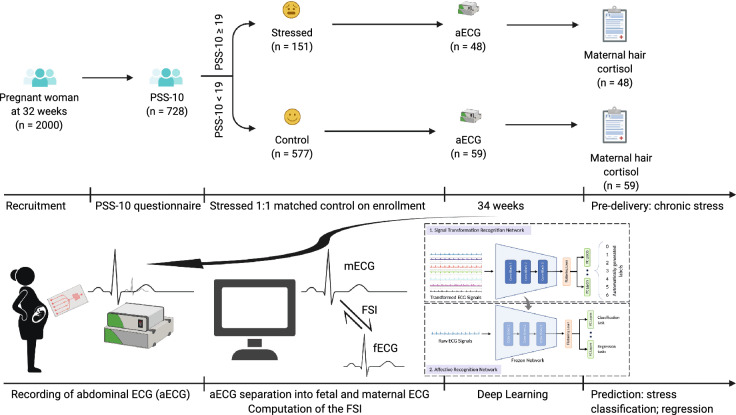
Summary of the approach: Prenatal Distress Questionnaire (PDQ) and Prenatal Stress Score (PSS-10) were determined in 32 weeks pregnant women classifying them as stressed group or matched controls. At 34 weeks, abdominal ECG (aECG) was recorded and prior to delivery, maternal hair was sampled for cortisol measurements reflecting chronic stress exposure over the past 2 months. The aECG was deconvoluted into fetal and maternal ECG (fECG, mECG) from which Fetal Stress Index (FSI) was computed, reflecting joint maternal and fetal chronic stress exposure. Deep Learning using a self-supervised learning framework ensued on aECG and mECG (fECG did not qualify due to signal quality) to detect stress group status (i.e., classification) and values of cortisol, FSI, PDQ, and PSS-10 (i.e., regression).

Here, we hypothesized that a DL approach to pattern recognition in maternal abdominal electrocardiograms (aECG) obtained in chronically stressed mothers and controls matched at enrolment at 32 weeks of gestation will detect chronic stress in mother-fetus dyads, i.e., a DL classification model (Fig. 1).

We validated the exposure to stress by psychological inventory, molecular and biophysical biomarkers including maternal hair cortisol and FSI, respectively. Then, we tested the correlation between these exposure measures and the aECG and maternal ECG (mECG) features captured by the DL pipeline, i.e., DL regression model. We implemented the DL pipeline using the recently established self-supervised learning (SSL) approach that provides emotional recognition from ECG^5,6^.

We tested two variants of SSL architecture, one trained on the generic ECG features for emotion recognition obtained from public datasets and another transfer-learned on a subset of the composite aECG (which includes fetal ECG, fECG) or mECG data. Our studies of the model’s performance in regression tasks and with or without the inclusion of the fetal ECG signal reveal a rich structure correlating to psychological, molecular, and biophysical biomarkers of maternal and fetal stress exposure at 34 weeks of gestation and at birth.

## Results

### Datasets: characteristics and differences

There were no differences in age between the cohorts of our and the public datasets and the total number of subjects; in the public dataset used for training there were 103 subjects compared to 107 in FELICITy dataset (Table 1). We refer the interested reader to the supplementary material for further details regarding the public datasets. We reported the clinical characteristics of the FELICITy cohort dataset originally in^1^. In the present expanded cohort the clinical characteristics remained almost unchanged. We reported them in Table S1 of^7^. Comparing the stressed group to control, with regard to 5-min Apgar scores, umbilical artery blood pH, white blood cell counts, birth weight, labor induction, and admission to NICU there were no differences. Cesarian delivery was found to be performed more frequently in the stressed group (p = 0.035).

**Table 1:** Demographic and dataset characteristics.

Dataset	AMIGOS	DREAMER	WESAD	SWELL	FELICITy
No. of participants	40	23	15	25	107
Female/male (count)	13/27	9/14	3/12	8/17	107/0
Age (years)	28.3 (21–40)	26.6 ± 2.7	27.5 ± 2.4	25 ± 3.25	33 ± 4
Duration (min.)	95	60	120	95	46
Sampling rate (Hz)	256	256	700	2048	900

A clear difference existed in the gender composition, albeit its impact on the model performance remains uncertain. ECG duration was more variable in the public dataset than in the FELICITy dataset and so was the sampling rate. However, it remains also uncertain whether this had any impact on the model performance, especially since all ECG was resampled at 256 Hz for the DL pipeline. It is possible that such variance in data quality and the composition of the participants made the model more robust, but this conjecture would need to be tested in future work. Within the FELICITy dataset, there was no difference in the gestational ages of the fetuses from control and stressed groups at the time of PPS-10 test (34.0 (33.3–35.0) versus 34.0 (32.6–34.9), p = 0.304), ECG recording (36.7 (35.2–37.6) versus 36.4 (35.3–37.4), p = 0.612) and delivery (39.9 (39.0–40.6) versus 39.5 (38.6–40.6), p = 0.148).

We tested if there is a relationship between PSS and FSI and whether it depends on fetal sex. We conducted a linear regression analysis of FSI predicted by PSS for each sex. The results were non-significant and including both male and female subjects did not change this finding: the adjusted R2 value was 0.006 (p = 0.181). We then asked if, perhaps, the relationship only exists among the stressed subjects. This hypothesis was also not validated: adjusted R2 value was 0.01 (p = 0.59).

We also studied the relationship between FSI and fetal sex. As we reported in^7^, FSI shows sex-specific patterns with regard to chronic exposure to stress. The differences in FSI due to stress exposure were seen in male fetuses (stressed: 0.30 ((− 0.18) − 0.61) versus control: − 0.13 ((− 0.45) − 0.31), median (interquartile range), p = 0.050), but not in female fetuses (stressed: 0.27 (± 0.84) versus control: 0.10 (± 0.55), mean (SD), p = 0.394). The sample size for this comparison was as follows: male: n = 43 stressed, n = 35 control; female: n = 22 stressed, n = 39 control.

We compared the model performance for detecting stressed mother-fetus dyads as well as predicting maternal hair cortisol, FSI, PDQ, and PSS values depending on two factors: the source of ECG (aECG, mECG) and the source of the trained model (learning from the FELICITy dataset—the first SSL approach, or transfer-learning from the public datasets—the second SSL approach) (Tables 2,  3).

**Table 2:** Detection of stressed mothers by self-supervised learning trained on the FELICITy and public datasets.

Source	Accuracy	F1 Score	Sensitivity	Specificity	PPV	NPV	AUROC
**FELICITy dataset**							
aECG	0.795 ± 0.023	0.777 ± 0.022	0.779 ± 0.031	0.809 ± 0.045	0.777 ± 0.039	0.812 ± 0.020	0.794 ± 0.022
mECG	0.931 ± 0.093	0.925 ± 0.101	0.924 ± 0.101	0.937 ± 0.087	0.926 ± 0.102	0.936 ± 0.086	0.931 ± 0.094
**Public datasets**							
aECG	0.936 ± 0.002	0.930 ± 0.003$^{*}$	0.926 ± 0.008$^{*}$	0.945 ± 0.004$^{*}$	0.935 ± 0.004$^{*}$	0.938 ± 0.006$^{*}$	0.936 ± 0.002$^{*}$
mECG	0.982 ± 0.003	0.980 ± 0.003$^{\#*}$	0.982 ± 0.004$^{\#*}$	0.982 ± 0.006$^{\#*}$	0.979 ± 0.007$^{\#}$	0.985 ± 0.003$^{\#}$	0.982 ± 0.002$^{\#*}$

$^{*}$Public versus FELICITy dataset, Mann Whitney U test.

$^\#$maternal ECG (mECG) versus abdominal ECG (aECG) within the same dataset, Mann Whitney U test.

Statistical significance at p < 0.025 accounting for two comparisons (using Bonferroni–Holm correction).

*PPV* positive predictive values, *NPV* negative predictive values, *AUROC* area under the receiver operating characteristic.

**Table 3:** Prediction of biomarkers by self-supervised learning on the FELICITy and public datasets.

Task	Source	R2 FELICITy dataset	R2 Public datasets
Cortisol	aECG	0.456 ± 0.053	0.801 ± 0.009$^{*}$
	mECG	0.743 ± 0.322	0.931 ± 0.006$^{\#*}$
FSI	aECG	0.362 ± 0.052	0.768 ± 0.018$^{*}$
	mECG	0.780 ± 0.274	0.946 ± 0.013$^{\#*}$
PDQ	aECG	0.408 ± 0.062	0.781 ± 0.019$^{*}$
	mECG	0.789 ± 0.302	0.961 ± 0.010$^{\#*}$
PSS	aECG	0.344 ± 0.072	0.761 ± 0.012$^{*}$
	mECG	0.780 ± 0.294	0.943 ± 0.009$^{\#}$

$^*$Public versus FELICITy dataset, Mann Whitney U test

$^\#$maternal ECG (mECG) versus abdominal ECG (aECG) within the same dataset, Mann Whitney U test

Statistical significance at p < 0.025 accounting for two comparisons (using Bonferroni–Holm correction).

*FSI* Fetal Stress Index, *PDQ* Prenatal Distress Score, *PSS* Perceived Stress Scale score.

### Identification of stressed mother-fetus dyads: DL classification task

Within the FELICITy dataset, the ECG source made no difference, but using the public dataset improved the F1 score, sensitivity, specificity and AUROC regardless of the ECG source (Table 2). In comparison to the FELICITy dataset, training on the public dataset while using aECG improved performance across all metrics except the accuracy. Accuracy was excellent overall and stood out as not being influenced by the ECG source or the origin of the trained model. Again in comparison to the FELICITy dataset, training on the public dataset while using mECG also improved performance overall, except accuracy, PPV, and NPV. This was because mECG in general boosted the performance regardless of how the model was trained—on the FELICITy or the public datasets. The best group classification performance overall, across all metrics, was achieved using the public dataset and mECG.

### Prediction of stress biomarkers: DL regression task

Recognizing the spread of PSS-10 scores, in the present study we also assessed the regression relationship between the scores and emotional recognition performance in our DL model (Table 3). The model performance results were similar for the regression analyses. We see overall similar improvements and best performance for all biomarkers when using mECG and the public dataset. When using the model trained on the FELICITy dataset, there was no difference in prediction for all biomarkers when using aECG or mECG. This suggests there is enough information in the mECG and the model trained on the FELICITy dataset. In contrast, using the model trained on the public dataset improved the performance regardless of the source of data, aECG or mECG.

For aECG on the FELICITy dataset, the model performed poorly for all biomarkers. Using mECG instead brought no significant improvement. When training on the public dataset, the performance improved on both aECG and mECG for cortisol, FSI, and PDQ, but not for PSS when using mECG, because it is already quite accurate when trained on the FELICITy dataset. In other words, the prediction of the PSS scores achieves highest performance when using the SSL pipeline trained on the FELICITy dataset and using mECG rather than the composite aECG, i.e., a signal containing maternal and fetal ECG combined.

For FSI and PDQ, it appears that the effect of the regression improvement by using the public dataset is dependent not on the biomarker, but on the data source, i.e., aECG versus mECG. This may be explained by the richer intrinsic structure of aECG compared to the uniquely maternal sourced mECG, which is better captured by the public dataset. The public dataset was also richer than the FELICITy dataset with regard to participants’ gender composition, ECG sampling rate and duration (Table 1).

**Figure 2:**
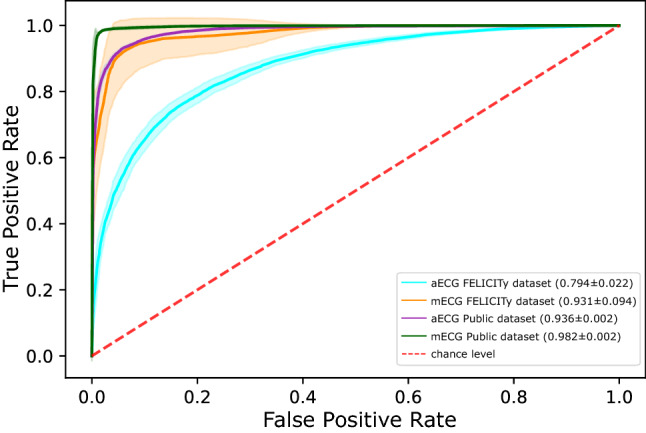
AUROC of SSL models trained on the public and FELICITy datasets to identify stressed and non-stressed mother-fetus dyads from aECG or mECG. Mean AUROC values are marked as solid lines and standard deviations across fivefolds are marked as shaded regions.

Overall, using the raw aECG decreases the model performance on both classification and regression. Identification of the effects of chronic stress and a highly accurate prediction of its effects on cortisol, FSI, PDQ, and PSS is possible from maternal ECG alone using the SSL model trained on the public dataset and using FELICITy dataset does not improve this performance neither for classification nor for regression. This is visualized in Fig. 2.

## Discussion

Chronic stress is one of the most common modifiers of fetal and postnatal development with lifelong lasting effects on health and no systematic prevention programs exist today^8,9^. The present findings provide a solution. First, confirming our hypothesis, we report a scalable and readily deployable approach using an SSL model of DL to identify chronically stressed mother-fetus dyads and predict their biochemical, biophysical, and psychological characteristics from a regular mECG with a high degree of accuracy. The excellent performance of the model trained on the public dataset suggests a high probability of generalizability of our findings to new data.

**Figure 3:**
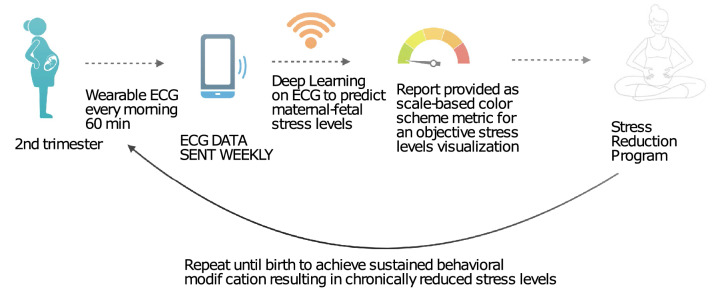
Real-world application of our AI model to reduce stress during pregnancy and prevent its long-term sequelae.

This is an important advance in early and non-invasive detection of chronic stress effects during pregnancy. The demonstration of mECG being sufficient translates into the ability of using conventional ECG devices which are widely available already. This will also enable wider utilization of ECG for studies of chronic stress effects on maternal, fetal, and postnatal health. Figure 3 demonstrates a possible deployment scenario made feasible by this work. For example, a logical next step would be to validate the present method in the daily life setting of pregnant women, as opposed to a clinical setting.

Another novel insight stems from two related observations. First, there was a high degree of accuracy in predicting individual characteristics of the mother-fetus dyads related to chronic stress (cortisol, FSI, PDQ, and PSS). Second, an exploration of the neural network’s latent space features suggests strongly that the entire ECG waveform structure is required and not only the temporal features of R–R peaks, i.e., heart rate variability (data not shown).

The deep neural network properties are important to consider for two reasons. First, there appears to be a rich intrinsic integrated information about these distinct physiological properties contained in ECG. This information is retained after the temporal order is destroyed by permutation of ECG waveforms as done in this work. To our knowledge, this is the first demonstration of such a relationship. Further research is needed to uncover the precise changes in the maternal and fetal ECG waveform characteristics which contribute to the excellent performance of the presented DL model. Second, most presently available wearables do not record continuous ECG, but, rather, use photoplethysmography (PPG) sensors to track heart rate triggered from the pulse waveform^10^. A new DL approach suggests that higher quality ECG signal can be derived from PPG using a generative adversarial network (GAN) architecture^11^. Such techniques^11^ have the potential to be used in present day wearables for identifying stress. However, at this point we restrict ourselves from conducting such an experiment as our dataset does not contain corresponding PPG recordings. Meanwhile, a next generation of wearables is capable of continuous on-body ECG monitoring^12^, while some readily available clinical-grade ECG trackers can be deployed for this purpose already^13^.

In^1^ we introduced the FSI as a novel biophysical biomarker of maternal-fetal stress memory using 74% of the since completed FELICITy cohort dataset. In the present study, we expand this dataset to its complete size of 173 participants and apply a novel DL method on raw ECG data. This stands in contrast to the more complex pipeline in the previous work^1^ which requires fECG extraction, R peak detection, and bPRSA computation to derive FSI. The two studies build upon each other as we establish here an approach to predict the more difficult to compute FSI metric from raw ECG data using the DL methodology.

The original designation of the DL algorithm as providing emotional recognition from ECG raises the question as to what stress we are detecting using the presented approach. Our DL model assumes that ECG contains implicit information about stress state, hence its designation as providing emotional recognition. The evidence we provide in this study is that this DL approach is also capable of predicting other types of stress memory captured by PSS, PDQ, cortisol and FSI. As we discuss above, our DL approach likely captures the effects of stress on ECG waveform and temporal properties. PSS and PDQ quantify stress exposure on an integrated psychological scale. We consider these metrics as capturing a combination of both the emotional response to stress and the (conscious) stress appraisal. FSI and cortisol quantify an emotional response to stress on the (unconscious) level of the autonomic nervous system. FSI quantifies entrainment of fHR by mHR, a fundamental property of weakly coupled nonlinear dynamical systems reflecting their stability in phase space. Hair cortisol reflects stress exposure over 3 months. We discuss this in^1^.

We found no direct relationship between PSS and FSI. This is not surprising considering the multifactorial nature of the interactions involved. We investigated this to an extent using causal inference in Figure 3 of^7^. As also reported in^7^, we observed the differences in FSI due to stress exposure in male, but not female fetuses. Sex-related differences in fetal heart rate variability metrics and the synchronization between maternal and fetal heart rates quantified by cross-sample-entropy have been reported in healthy term pregnancies^14^ and support the present findings. The sex-specific difference in maternal-fetal stress imprint on the autonomic nervous system quantified by FSI warrants further study.

Our study has limitations. First, the datasets are relatively small with less than 200 subjects in both the public and FELICITy datasets, yet the model performance has shown satisfactory stability. Also, these datasets are the largest known to us so far to permit such investigation. Second, we abstained from complicating our model with the addition of ancillary features such as BMI which indeed was significantly different in our study between both groups. Overall, the groups’ demographic data differed with regard to clinical and socioeconomic characteristics: university degree, income, smoking status, BMI, assisted reproductive techniques, planned pregnancy, gestational diabetes and cesarean delivery rate. These differences may represent the causes of chronic maternal stress or contribute to stress over time therefore being significantly more frequent in the stressed group. It has been suggested that a bias may be added with such an approach that results from introducing unintended confounders in the causal inference sense, e.g., BMI may interact with ECG features or other features that interact with ECG in ways we don’t know and the results may be biased or even meaningless as a result. Third, in contrast to the current black-box DL approach, gauging maternal-fetal interactions using FSI provides unique insights into the relationship between two biological systems, mother and fetus. In our previous publication^1^, we hypothesized an “over-sensitization” of stressed fetuses’ maturation of the autonomic nervous system in contrast to the controls. Fetal autonomic nervous system is very sensitive to maternal stress^8^. Thus, FSI may capture a predisposition for a later mental disorder. Consequently, FSI may serve as a novel biomarker to detect the effects of chronic prenatal stress early in utero. Our DL approach predicted FSI with high accuracy. Such strategy will aid in timely identification of infants for early intervention programs^15^. Fourth, in the present study we did not account for fetal sleep states. Doing so would be an important future research question. The key reason we have not made this adjustment stems from the fact that such an approach would considerably complicate the data recording: we would need to conduct a transabdominal ultrasound on the fetus to infer the behavioral state. Such studies have been done, but, especially in the stressed pregnant population, this would seem to be a problematic addition to the protocol. Taking 40 min of recording, as done in the present work, had the benefit of automatically containing at least one full pair of active (low-voltage/high-frequency brain electrical activity) and quiet (high voltage/low frequency) sleep states. Moreover, fetuses spend some 95% of their time in sleep^16^. Consequently, the probability of capturing the fetus in an awake state was very low. In summary, we suggest that no additional adjustment for fetal sleep/wake states on the present data is required.

In conclusion, maternal-fetal early-life stress and its molecular and biophysical characteristics can be predicted with very good accuracy and reproducibility from regular ECG using a scalable SSL deep learning approach.

## Methods

### FELICITy study

Ethics approval was obtained from the Committee of Ethical Principles for Medical Research at the TUM (registration number 151/16S; ClinicalTrials.gov registration number NCT03389178). All methods were performed in accordance with the relevant guidelines and regulations. We obtained informed consent for study participation from each subject.

**Figure 4:**
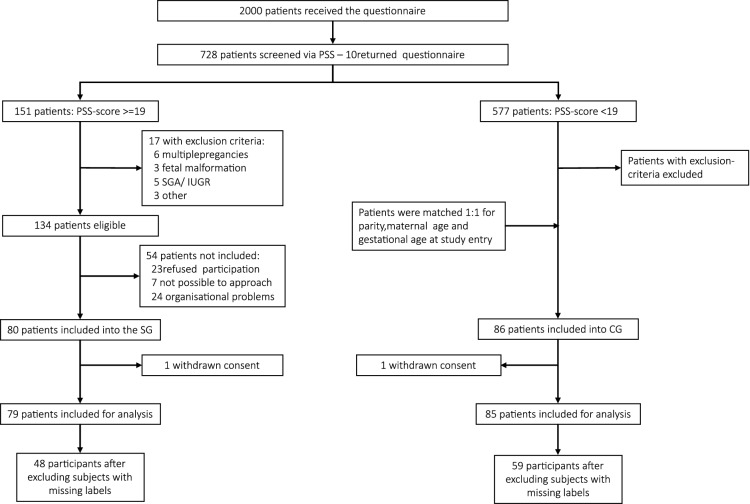
Recruitment flow chart for the FELICITy dataset: from screening to deep learning.

The complete experimental design can be found in^1^. Briefly, in this prospective study, stressed mothers were matched with controls 1:1 for parity, maternal age, and gestational age at study entry. Recruited subjects were between 18 and 45 years of age, and were in their third trimester. The study ran for 22 months from July 2016 until May 2018, and subjects were selected from a cohort of pregnant women followed in the Department of Obstetrics and Gynecology at “Klinikum rechts der Isar” of the Technical University of Munich (TUM). This is a tertiary center of Perinatology located in Munich, Germany, which serves  2000 mothers/newborns per year. Figure 4 presents the recruitment flowchart for this dataset and the use of data in this study. Four exclusion criteria were applied, namely (a) serious placental alterations defined as fetal growth restriction according to Gordijn et al.^17^; (b) fetal malformations; (c) maternal severe illness during pregnancy; (d) maternal drug or alcohol abuse.

The Cohen Perceived Stress Scale questionnaire was administered to gauge chronic non-specific stress exposure (PSS-10)^18^. PSS-10 $\ge$ 19 categorized subjects as stressed, as established^1^. We applied inclusion- and exclusion criteria following returning the questionnaires. When a subject was categorized as stressed, the next screened participant matching for gestational age at recording with a PSS-10 score < 19 was entered into the study as control. In addition to PSS-10, the participants received the German Version of the “Prenatal Distress Questionnaire” (PDQ) containing 12 questions on pregnancy related fears and worries regarding pregnancy related changes of the body weight and troubles, child’s health, delivery and pregnancy’s impact on the women’s relationship.

For recording ECG, we organized the clinical setting to be as similar as possible for all study participants. We performed the recordings in all women in supine half left recumbent position, usually at the same time of day (afternoon). A transabdominal ECG (aECG) recording with a sampling rate of 900 Hz and a duration of at least 40 min was performed two and a half weeks after screening. The AN24 (GE HC/Monica Health Care, Nottingham, UK) was used. We calculated the signal quality index (SQI)^19^ for aECG, in 1-s windows, and subsequently discarded segments with an SQI of lower than 0.5. Using the fetal and maternal ECG deconvolution algorithm SAVER^19^, we extracted fetal ECG (fECG) and maternal ECG (mECG).

We utilized SQI to discard the noisy data resulting in the averaged duration of mECG and aECG of 46.07 ± 8.74 min, whereas the average duration of fECG was 3.25 ± 7.83 min. Due to its short duration, we could not utilize the extracted fECG data to train our self-supervised model and continued with aECG and mECG signals only. However, please note that aECG signal does represent a composite signal containing fECG and mECG, so DL on this signal tells us something about the fECG features. We refer to the resulting ECG dataset containing aECG and mECG as the FELICITy dataset.

We detected the fetal R-peaks and the maternal R-peaks separately from the respective fECG and mECG signals. The fetal- and maternal RR interval time-series were subsequently derived from the fetal and maternal R-peaks. We then calculated the mean fetal heart rate (fHR) and mean maternal heart rate (mHR) values.

Upon delivery of the baby, we recorded the clinical data including birth weight, length, and head circumference, pH, and Apgar score. Maternal cortisol was assessed using established methodology^20–22^.

### Bivariate phase rectified signal averaging

To analyze the relationship between two signals recorded synchronously, mHR and fHR, we use the bivariate phase rectified signal averaging (BPRSA) method^23^. This method extends the “monovariate” PRSA method proposed for the analysis of fHR^24,25^.

The two signals in question in this study are the mHR (trigger signal) and the fHR (target signal). The BPRSA algorithm operates by first detecting a number of anchor points *A*, defined as decreases in mHR. Next, for the detected set of *A*, we interpolate the fHR with a sampling rate of 900 Hz to match the maternal ECG. We then detect the time of the anchor points in fHR, which we denote by $A'$. Then, around each anchor point $A'$ in fHR, a window of length (2*L*) is selected. In this paper, we set *L* = 9000, resulting in a window of 20 s. Next, by aligning the anchor points, we obtain phase-rectified segments. The resultant segments are then averaged to obtain BPRSA signal *X*. Consequently, we can interpret defections in *X* as coupling between mHR and fHR. Lastly, *X* is quantified within specific windows before and after the center of *X*. Accordingly, the designated windows are characterized as *L* + *S*1 to *L* + *S*2, and *L* + *S*2 to *L* + *S*1, where *S*1 and *S*2 are the indices used for this quantification step. We set *S*1 = 1350 and *S*2 = 2250, which results in windows of 1.5 and 2.5 s, given our sampling rate of 900 Hz.

Fetal stress index (FSI) is a parameter defined to analyze the coupling between mHR and fHR using the BPRSA. This index is defined as the difference between the means of the two windows mentioned above, as follows:

1 $$\begin{aligned} FSI = \frac{1}{S2-S1} \sum _{i=L+S1}^{L+S2}X(i) - \frac{1}{S2-S1} \sum _{i=L-S2}^{L-S1} X(i), \end{aligned}$$

where index *L* at the center of *X* corresponds to our anchor definition (within the maternal RR intervals). Accordingly, the response of the fetus on mHR decreases is measured by FSI.

### Representation learning

**Figure 5:**
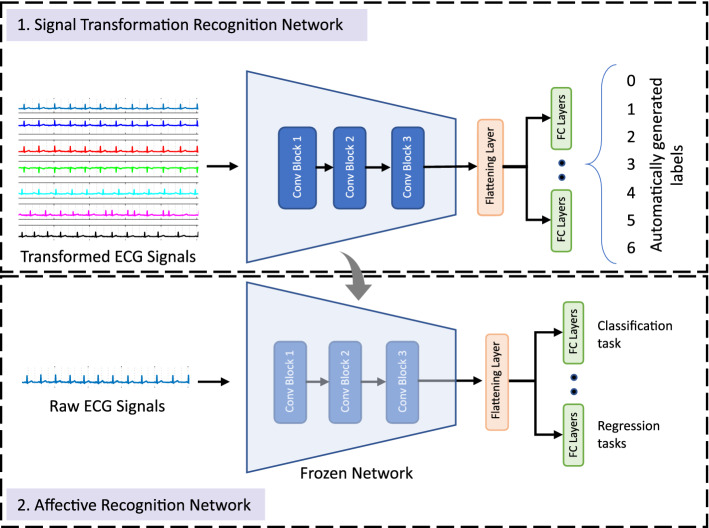
Our deep learning approach using a self-supervised learning framework.

We utilized an established self-supervised learning framework^5,6^ to learn robust representations from our collected ECG data, which were further used to classify the level of stress, as well as to perform regression analyses. The framework consisted of two stages of learning, the first stage consisted of learning ECG representations and the second stage consisted of learning affect attributes from the learned representations (see Fig. 5).

#### Learning ECG representations

We utilized a multi-task convolutional architecture, henceforth referred to as the ‘transformation recognition network’, which consists of three convolutional blocks. Each block consists of two 1D convolution layers with leaky rectified linear unit (ReLU) activation functions, followed by a max pooling layer. Following the convolutional layers, a global max pooling is used. This is finally followed by the several parallel fully connected (FC) layers. We applied dropouts to reduce the possibility of overfitting. A detailed description of this network’s architecture is given in supplementary material.

In order to learn the ECG representation, the model was trained in a self-supervised manner. Automatic labels were generated through the following transformations:

1. Noise addition: Random Gaussian noise is added to the raw ECG signal.
2. Scaling: The magnitude of the original ECG is scaled.
3. Negation: The original ECG signal is flipped vertically.
4. Temporal Inversion: The original ECG signal is flipped horizontally.
5. Permutation: The raw ECG signal is first divided into smaller segments of equal length, which are then randomly shuffled across the time axis.
6. Time-warping: ECG signals are first divided into smaller segments similar to the permutation operation, These segments are then stretched or squeezed across the time axis.

The parameters of the above-mentioned transformations were derived from our previous work^6^. Next the transformed signals were stacked randomly to create the input matrix for the self-supervised network, while the corresponding labels of the transformations were stacked, in a similar order to the inputs, to create the output labels. Each of these transformation labels are used as an output to one of the FC layers to construct a multi-task network.

#### Learning affect

In the second stage, affective attributes were learned using the learned ECG representations obtained from the self-supervised network. In this stage we classified stress followed by regression analysis of maternal hair cortisol, FSI, PDQ, and PSS values. The affect recognition network contains the similar convolutional layers as those used in the self-supervised network, followed by fully connected layers. The weights of the convolution layers are transferred from the signal transformation recognition network and kept frozen, and only the fully connected layers are trained. Detailed descriptions of the architectures are mentioned in the supplementary material.

### Training

#### Approaches

In order to explore the generalizability of the self-supervised method, we tackled this task in two different ways. Our first approach was to use FELICITy dataset and train the framework from scratch. As the second approach, we utilized four publicly available datasets to train the signal transformation network for learning ECG representations, followed by using FELICITy dataset to perform affect recognition by training the fully connected layers of the second network. The details of these two approaches are mentioned below.

*First approach—learning from FELICITy dataset* As mentioned above, in our first approach, we utilized FELICITy dataset to train the self-supervised network consisting of both the signal transformation recognition network responsible for learning to extract ECG representations, as well as the fully connected layers of the affect recognition network.

*Second approach—transfer learning from public datasets* In order to explore the generalizability of the self-supervised learning, we used four publicly available datasets namely, AMIGOS^26^, DREAMER^27^, SWELL^28^, and WESAD^29^ to train the signal transformation recognition network, i.e., learn ECG representations. Next, we transferred the weights of the network to the affect recognition network where we utilized FELICITy dataset and collected labels to train the fully connected layers of the network so that stress can be classified and factors such as maternal hair cortisol, FSI, PDQ, and PSS values can be regressed. A description of the public datasets is provided in supplementary material.

#### Implementation details

We performed minimal pre-processing on the raw data. We re-sampled ECG signals to a sampling frequency to 256 Hz, followed by segmentation into 10-s windows as proposed by^6^. Next, to remove the noisy parts of aECG and mECG data, we utilized the SQI values available with the segments. To this end, SQI < 0.5 were discarded. This resulted in removing approximately 4.1% of total acquired data with a standard deviation of 8.8. In other words, approximately 50 min (46.07 ± 8.74) of ECG data from each participant were used.

We divided our whole dataset into training and test sets using a 5-fold cross-validation technique as follows. To create the training and test sets, we randomly divided each person’s data into five equal parts, where four parts were selected for training, and the one part was used for testing. The process was repeated five times.

The hyper-parameters for the self-supervised model are the same as those use in our earlier work^6^. An Adam optimizer was used to train the models with a learning rate of 0.001 and a batch size of 128. A binary cross-entropy loss was used for the classification task and mean absolute error loss was used for the regression tasks.

The fetal and maternal ECG and HR extraction algorithms were carried out in Matlab R2016a. The SSL DL pipeline was implemented using NVIDIA GeForce RTX 2070 GPU in TensorFlow 1.14, and is publicly available at: https://github.com/pritamqu/SSL-ECGv2.

### Statistical analyses

We used the Shapiro–Wilk test to evaluate for normal distribution. Medians and interquartile ranges were reported for skewed distributions, while the means and standard deviations are reported for Gaussian distributions. Where data are categorical, we present the absolute and relative frequencies. Groups are compared using t-test for independent samples, Mann-Whitney U test, and Pearson Chi-squared test.

All of the statistical tests were performed two-sided with statistical significance considered at p < 0.05. The Bonferroni–Holm correction was used to adjust for multiple comparisons. To estimate the predictive performance of the quantitative variables for the presence of PS, receiver operating characteristics (ROC) analyses were carried out. Linear regression analyses were conducted to quantify the model performance in the regression tasks, expressed as R2 (see Table 3), Mean Average Error (MAE), and Root Mean Squared Error (RMSE) (see Table S1). All analyses were done with Python v3.6 Scipy library.

## Supplementary Information

Supplementary Information.

## References

[1] Lobmaier SM (2020). Fetal heart rate variability responsiveness to maternal stress, non-invasively detected from maternal transabdominal ECG. Arch. Gynecol. Obstet..

[2] LeCun Y, Bengio Y, Hinton G (2015). Deep learning. Nature.

[3] Sarkar, P. *et al.* Classification of cognitive load and expertise for adaptive simulation using deep multitask learning. In *8th IEEE International Conference on Affective Computing and Intelligent Interaction*, 1–7 (2019).

[4] Ross K (2019). Toward dynamically adaptive simulation: Multimodal classification of user expertise using wearable devices. Sensors.

[5] Sarkar, P. & Etemad, A. Self-supervised learning for ECG-based emotion recognition. In *IEEE International Conference on Acoustics, Speech and Signal Processing*, 3217–3221 (2020).

[6] Sarkar P, Etemad A (2020). Self-supervised ECG representation learning for emotion recognition. IEEE Trans. Affect. Comput..

[7] Zimmermann, P. *et al.* Prenatal stress perturbs neonatal iron homeostasis in a sex-specific manner. arXiv preprint arXiv:2105.12809 (2021). 10.1038/s41598-022-13633-z PMC9167276 35662279

[8] Frasch MG (2018). Non-invasive biomarkers of fetal brain development reflecting prenatal stress: An integrative multi-scale multi-species perspective on data collection and analysis. Neurosci. Biobehav. Rev..

[9] Desplats P, Gutierrez AM, Antonelli MC, Frasch MG (2019). Microglial memory of early life stress and inflammation: Susceptibility to neurodegeneration in adulthood. Neurosci. Biobehav. Rev..

[10] Gonçalves H, Pinto P, Silva M, Ayres-de Campos D, Bernardes J (2016). Electrocardiography versus photoplethysmography in assessment of maternal heart rate variability during labor. Springerplus.

[11] Sarkar, P. & Etemad, A. Cardiogan: Attentive generative adversarial network with dual discriminators for synthesis of ECG from PPG. In *Proceedings of the AAAI Conference on Artificial Intelligence* **35**, 488–496 (2021).

[12] Jeong H, Rogers JA, Xu S (2020). Continuous on-body sensing for the covid-19 pandemic: Gaps and opportunities. Sci. Adv..

[13] Herry CL, Soares HM, Schuler-Faccini L, Frasch MG (2021). Machine learning model on heart rate variability metrics identifies asymptomatic toddlers exposed to zika virus during pregnancy. Physiol. Meas..

[14] Gonçalves H, Fernandes D, Pinto P, Ayres-de Campos D, Bernardes J (2017). Simultaneous monitoring of maternal and fetal heart rate variability during labor in relation with fetal gender. Dev. Psychobiol..

[15] Antonelli MC (2021). Early biomarkers and intervention programs for the infant exposed to prenatal stress. Curr. Neuropharmacol..

[16] Rao N, Keen A, Czikk M, Frasch M, Richardson BS (2009). Behavioural state linkage in the ovine fetus near term. Brain Res..

[17] Gordijn S (2016). Consensus definition of fetal growth restriction: A Delphi procedure. Ultrasound Obstet. Gynecol..

[18] Cohen S, Kamarck T, Mermelstein R (1983). A global measure of perceived stress. J. Health Soc. Behav..

[19] Li R, Frasch MG, Wu H-T (2017). Efficient fetal-maternal ECG signal separation from two channel maternal abdominal ECG via diffusion-based channel selection. Front. Physiol..

[20] Cooper GA, Kronstrand R, Kintz P (2012). Society of hair testing guidelines for drug testing in hair. Forens. Sci. Int..

[21] Iglesias S (2015). Hair cortisol: A new tool for evaluating stress in programs of stress management. Life Sci..

[22] Gonzalez D (2019). Hair cortisol measurement by an automated method. Sci. Rep..

[23] Bauer A, Barthel P, Müller A, Kantelhardt J, Schmidt G (2009). Bivariate phase-rectified signal averaging—A novel technique for cross-correlation analysis in noisy nonstationary signals. J. Electrocardiol..

[24] Lobmaier S (2012). Phase-rectified signal averaging as a new method for surveillance of growth restricted fetuses. J. Maternal-Fetal Neonatal Med..

[25] Lobmaier SM (2016). Phase-rectified signal averaging method to predict perinatal outcome in infants with very preterm fetal growth restriction—A secondary analysis of truffle-trial. Am. J. Obstet. Gynecol..

[26] Correa JAM, Abadi MK, Sebe N, Patras I (2018). Amigos: A dataset for affect, personality and mood research on individuals and groups. IEEE Trans. Affect. Comput..

[27] Katsigiannis S, Ramzan N (2017). Dreamer: A database for emotion recognition through EEG and ECG signals from wireless low-cost off-the-shelf devices. IEEE J. Biomed. Health Inform..

[28] Koldijk, S., Sappelli, M., Verberne, S., Neerincx, M. A. & Kraaij, W. The swell knowledge work dataset for stress and user modeling research. In *Proceedings of the 16th International Conference on Multimodal Interaction*, 291–298 (2014).

[29] Schmidt, P., Reiss, A., Duerichen, R., Marberger, C. & Van Laerhoven, K. Introducing wesad, a multimodal dataset for wearable stress and affect detection. In *Proceedings of the 20th ACM International Conference on Multimodal Interaction*, 400–408 (2018).

