# Supplementary material for: Detection of maternal and fetal stress from the electrocardiogram with self-supervised representation learning
Source: Sci Rep. 2021 Dec 17;11:24146. doi: 10.1038/s41598-021-03376-8 (PMC8683397; doi:10.1038/s41598-021-03376-8)
Supplement: Supplementary file 1 — Supplementary Information. [file 41598_2021_3376_MOESM1_ESM.pdf]

Supplementary Materials

Pseudocode

Algorithm 1 Self-supervised pretraining

```
# model: randomly initialized CNN
# loss_coeff: weights of each loss
# optimizer: Adam optimizer
# calculate_loss: binary cross entropy loss

# training
for epoch in range(start_epoch, end_epoch):
   # sample a batch of raw ecg signals
   for batch in enumerate(train_dataloader):
      # perform transformation and generate pseudo labels
      train_batch, train_plabels = transformation(batch)
      # shuffle data
      train_batch, train_plabels = shuffled(train_batch, train_plabels)
      # fit data to model
      logits = model(train_batch)
      # calculate individual task losses
      losses = calculate_loss(train_plabels, logits)
      # calculate total loss
      total_loss = get_weighted_loss(loss_coeff, losses)
      # back propagate the loss
      optimizer.minimize(total_loss)
```

Algorithm 2 Stress recognition finetuning

```
# model: pretrained model from ssl pretraining + newly added fc layer
# calculate_loss: classification or regression loss
# optimizer: Adam optimizer

# training
for epoch in range(start_epoch, end_epoch):
   # sample a batch of ecg and labels
   for train_batch, train_labels in enumerate(train_dataloader):
      # fit the data
      logits = model(train_batch)
      # calculate loss
      losses = calculate_loss(train_labels, logits)
      # back propagate
      optimizer.minimize(total_loss)
```

Network architectures

We utilize a popular convention to describe the CNN architectures. For example, cks2-f denotes a convolution layer with kernel size 1 ×k, stride 2, and f number of filters. mp8-s2 denotes a max-pool layer with a filter size of 8 and a stride 2. fcN denotes a fully connected layer with N hidden nodes. We utilize leaky-ReLU activation functions in all the convolution and fully connected layers, except the last layers, where sigmoid activation functions are used for the classification and recognition networks, and direct logits are extracted during the regression tasks. Finally, P×[fcN] indicates P number of parallel branches in the multi-task networks. Using this convention, the details of our models are given below.

Signal Transformation Recognition Network:

`c32s2-32, c32s2-32, mp8-s2, c16s2-64, c16s2-64, mp8-s2, c8s2-128, c8s2-128, global-max-pool, 7 × [fc128-dropout, fc128-dropout, fc1].`

Affect Recognition Network (classification):

`c32s2-32, c32s2-32, mp8-s2, c16s2-64, c16s2-64, mp8-s2, c8s2-128, c8s2-128, global-max-pool, fc512, fc512, fc1.`

Affect Recognition Network (regression):

`c32s2-32, c32s2-32, mp8-s2, c16s2-64, c16s2-64, mp8-s2, c8s2-128, c8s2-128, global-max-pool, 4 × [fc512, fc512, fc512, fc512, fc1]`

Prediction of stress biomarkers: DL regression task

In addition to Table 3, we further calculate mean absolute error (MAE) and root mean square error (RMSE) of our self-supervised framework in predicting cortisol, FSI, PDQ, and PSS (Table S1).

Supplementary Table S1. MAE and RMSE values for prediction of biomarkers using self-supervised learning, on the FELICITy and public datasets.

Task	Source	FELICITy dataset		Public datasets	
		MAE	RMSE	MAE	RMSE
Cortisol	aECG	37.832 ± 3.284	66.627 ± 3.209	$16.081 \pm 0.542^{*}$	$40.298 \pm 0.903^{*}$
	mECG	16.445 ± 16.068	40.621 ± 23.662	$6.676 \pm 0.334^{\#*}$	$23.729 \pm 0.977^{\#*}$
FSI	aECG	0.307 ± 0.025	0.541 ± 0.022	$0.114 \pm 0.005^{*}$	$0.326 \pm 0.013^{*}$
	mECG	0.111 ± 0.138	0.282 ± 0.164	$0.027 \pm 0.004^{\#*}$	$0.157 \pm 0.018^{\#*}$
PDQ	aECG	3.215 ± 0.316	5.648 ± 0.290	$1.158 \pm 0.057^{*}$	$3.436 \pm 0.151^{*}$
	mECG	1.189 ± 1.624	2.873 ± 1.984	$0.277 \pm 0.045^{\#*}$	$1.438 \pm 0.187^{\#*}$
PSS	aECG	3.851 ± 0.410	6.307 ± 0.335	$1.409 \pm 0.047^{*}$	$3.808 \pm 0.093^{*}$
	mECG	1.438 ± 1.772	3.177 ± 2.016	$0.423 \pm 0.039^{\#*}$	$1.851 \pm 0.161^{\#*}$

* Public versus FELICITy dataset, Mann Whitney U test.

$^{\#}$ Maternal ECG (mECG) versus abdominal ECG (aECG) within the same dataset, Mann Whitney U test.

Statistical significance at $p < 0.025$ accounting for two comparisons (using Bonferroni-Holm correction).

FSI: Fetal Stress Index, PDQ: Prenatal Distress Score, PSS: Perceived Stress Scale score

MAE: Mean absolute error, RMSE: Root Mean Squared Error

Description of Public Datasets

The key metrics of each dataset (AMIGOS[1], DREAMER[2], SWELL[3], and WESAD[4]) are summarized in Table 1 and are outlined in more detail below. It should be noted that all the public datasets contain ECG data and corresponding emotional ground truth labels. However, the emotional labels were not used in this study given the use of our self-supervised approach with automatically generated labels.

AMIGOS[1]:

This dataset comprises ECG and emotional labels from 40 participants. Participants were asked to watch different video clips (total 16) in order to elicit their emotional states. Shimmer ECG sensors[5] were used to record ECG at a sampling rate of 256 *Hz*. Finally, subjective arousal and valence scores were recorded on a scale of 1 to 9 at the end of each session.

DREAMER[2]:

The DREAMER dataset comprises data from 23 participants. The emotional responses were elicited by watching emotional video clips. The clips induced different emotions such as amusement, calmness, anger, excitement, disgust among others. Similar to AMIGOS, DREAMER was also collected using Shimmer ECG sensors[5] at a sampling rate of 256 *Hz*. At the end of each session Self-Assessment Manikins (SAM) were used to record arousal and valence scores on a scale of 1 to 5.

SWELL[3]:

25 participants comprised this dataset, where ECG data and affect scores were collected as participants performed different day-to-day office jobs, for example preparing reports, making presentations, and others. TMSI MOBI[6] devices were used in this study to collect ECG signals at a sampling rate of 2048 *Hz*. Finally, self-reported affect scores were collected on a scale of 1 to 9 at the end of each session.

WESAD[4]:

17 participants comprised the WESAD dataset. A RespiBAN Professional[7] sensor was used to collect ECG at a sampling rate of 700 *Hz*. Participants went through several tasks in order to elicit their emotional states, for

example, watching funny video clips during amusement condition, performing arithmetic tasks under stressed condition, reading magazines under normal conditions, and others. Finally, the Positive and Negative Affect Schedule (PANAS) scheme was used to collect emotional ground truth labels.

References

1. Correa, J. A. M., Abadi, M. K., Sebe, N. & Patras, I. Amigos: A dataset for affect, personality and mood research on individuals and groups. *IEEE Transactions on Affect. Comput.* (2018).
2. Katsigiannis, S. & Ramzan, N. Dreamer: A database for emotion recognition through eeg and ecg signals from wireless low-cost off-the-shelf devices. *IEEE J. Biomed. Heal. Informatics* **22**, 98–107 (2017).
3. Koldijk, S., Sappelli, M., Verberne, S., Neerincx, M. A. & Kraaij, W. The swell knowledge work dataset for stress and user modeling research. In *Proceedings of the 16th International Conference on Multimodal Interaction*, 291–298 (2014).
4. Schmidt, P., Reiss, A., Duerichen, R., Marberger, C. & Van Laerhoven, K. Introducing wesad, a multimodal dataset for wearable stress and affect detection. In *Proceedings of the 20th ACM International Conference on Multimodal Interaction*, 400–408 (2018).
5. Shimmer ecg. [Online]. Available: http://www.shimmersensing.com/products/shimmer3-ecg-sensor. [Accessed: 2020-09-17].
6. Tmsi-mobi. [Online]. Available: https://www.tmsi.com/products/mobi/. [Accessed: 2020-09-17].
7. Respiban professional. [Online]. Available: https://www.biosignalsplux.com/index.php/respiban-professional. [Accessed: 2020-09-17].
